# Effectiveness of Double-Hit Model (Post-Weaning Social Isolation and NMDA Receptor Antagonist) in the Development of Schizophrenic like Symptoms on Rodents: A Protocol for a Systematic Review

**DOI:** 10.3390/neurosci3010009

**Published:** 2022-02-09

**Authors:** Khanyiso Bright Shangase, Thabo Magwai, Fredrick Otieno Oginga, Khethelo Richman Xulu, Thabisile Mpofana

**Affiliations:** 1 Department of Human Physiology, School of Laboratory Medicine and Medical Science, College of Health Science, University of KwaZulu-Natal, Durban 4041, South Africa; thibos.thabo@gmail.com (T.M.); 221017678@stu.ukzn.ac.za (F.O.O.); xuluk2@ukzn.ac.za (K.R.X.); mpofana@ukzn.ac.za (T.M.); 2 National Health Laboratory Service, Department of Chemical Pathology, University of Kwa-Zulu Natal, Durban 4041, South Africa; 3 Developing Research, Innovation, Localisation and Leadership in South Africa (DRILL), School of Laboratory Medicine and Medical Sciences, College of Health Science, University of Kwazulu-Natal, Durban 4041, South Africa

**Keywords:** schizophrenia, animal model, social isolation, non-competitive NMDA receptor antagonist

## Abstract

Background: Schizophrenia is a heterogeneous neuropsychiatric disorder, categorized by positive, negative, and cognitive symptoms. In trying to improve the diagnosis and treatment of schizophrenia, researchers have turned to “dual hit” models of schizophrenia that are able to reproduce all symptoms of the disorder. The main objective of this protocol is to present a transparent process on how we plan to review the existing international literature on the effectiveness of “dual hit” models used to induce schizophrenia on rodents. Methods: Literature search strategies will be developed using medical search headings (MeSH). The MEDLINE (PubMed), EMBASE, and Google Scholar databases will be used to search for electronically published studies. We will search for studies involving inducing schizophrenic symptoms using “dual hit” rodent models (post-weaning social isolation and NMDA receptor antagonist). Studies will be screened by titles, abstracts, keywords, and synonyms followed by identifying the full-text articles. All studies that will pass quality assessment will be included. Data will be extracted by two authors independently and in duplicate from each eligible study to ensure that there is consistency between reviews. If the design and comparator are sufficiently homogenous for all studies, a meta-analysis will be conducted using a random-effect model. Discussion: The results of this review will contribute to the development of new “dual hit” models that will be able to characterize schizophrenia symptoms better. It will also shed light to researchers on new developments that need to be made in improving animal models of schizophrenia.

## 1. Introduction

Schizophrenia is a devastating neuropsychiatric disorder, characterised by intense disruptions in thinking which affect perception, language and the sense of self [1]. Global prevalence cases of schizophrenia increased from 13.1 million in 1990 to 20.9 million in 2016, with 14.8 million of these cases in the 25–54 years age group [2]. Reduced cholinergic firing, dopaminergic deregulation, hypo-function of NMDA receptors and dysfunction of GABAergic activity have been observed in schizophrenic patients. Symptoms are classically divided into three categories: positive, negative, and cognitive [3]. Although available antipsychotic treatment is highly effective in reducing positive symptoms, they have limited efficacy for negative and cognitive symptoms. Most animal models of schizophrenia have the tendency of replicating aspects of positive symptoms of schizophrenia, thus neglecting the important aspects of negative and cognitive symptoms [4]. Drug-induced models (ketamine, PCP, and MK-801) have been commonly used to replicate negative symptoms, while the developmental model (post-weaning social isolation) has been used to produce positive symptoms [5,6,7]. NMDA receptor antagonists influence neurodegeneration, excitotoxicity and apoptosis of mature corticolimbic pyramidal cells [8]. PCP, ketamine, and MK-801 are commonly used as selective NMDA receptor antagonists. A previous study on normal human volunteers showed that even at low doses, PCP produced psychotic symptoms, speech poverty and social withdrawal, which resemble negative symptoms of schizophrenia [9]. Social isolation is the lack of social contacts, interactions and relationships that have all been proven to impair cognitive ability and that can lead to several mental diseases such as schizophrenia [10,11,12]. Different animal models used to model combined heterogeneous psychiatric disorders are obviously treasured preclinical tools that are used to study the neurobiological basis of the disorder [4]. Better understanding of molecular and neuropathological mechanisms underlying schizophrenia will increase the chances of finding new therapeutic targets. This process requires formulations and the use of robust and reliable animal (rodent) models of schizophrenia using behaviours with translational significance and predictive validity. Currently, there is no single rodent model that fully reproduces the different schizophrenia symptom profiles; therefore, researchers have started to investigate “dual hit” models in rodents in order to produce more broad and robust deficits. The aim of this systematic review is to evaluate the effectiveness of a rodent “dual hit” model (post-weaning social isolation and NMDA receptor antagonist) of schizophrenia by:

- Investigating the production and severity of positive symptoms;
- Investigating the production and severity of negative and cognitive symptoms;
- Investigating the response to treatment (antipsychotics) of rodents with a “dual hit” model of schizophrenia.

**Figure 1:**
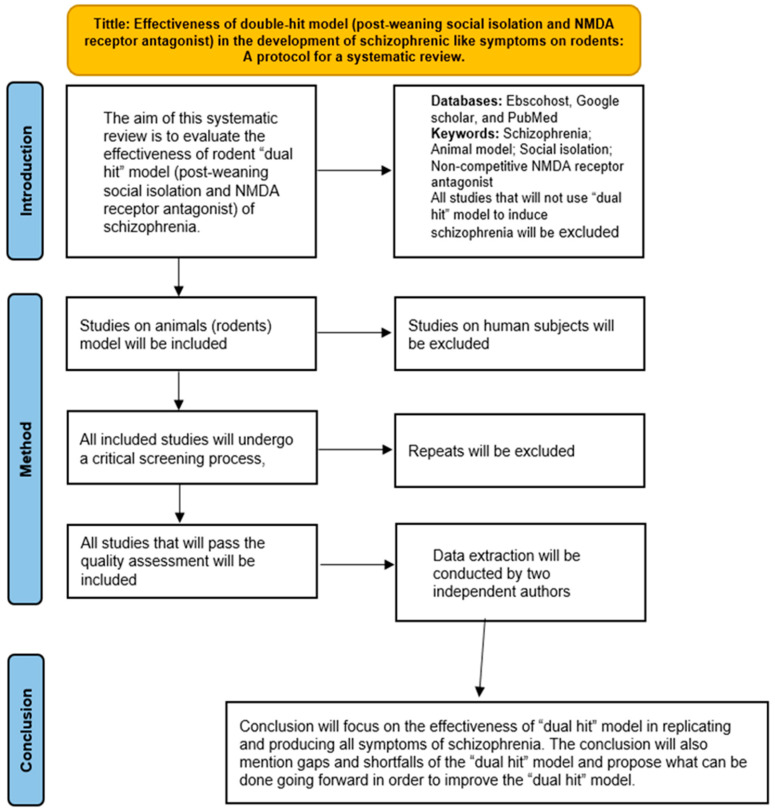
Flow diagram showing the summary of the protocol.

This systematic review is registered at the International Prospective Register of Systematic reviews (PROSPERO) (CRD42021247585). We will disclose any deviations from this protocol, and we will update the PROSPERO record accordingly. Figure 1 shows the summary of the protocol in a diagram form.

## 2. Methodology

This systematic review was developed according to the preferred reporting items for systematic review and meta-analysis protocols (PRISMA-P) [13].

### 2.1. Condition

The condition is schizophrenia as describe by authors using the following:

Population: we will focus on rodent animal models and on studies that used a “dual hit” model (post-weaning social isolation and NMDA receptor antagonist) to induce schizophrenia on rodents. We will exclude studies that only used post-weaning social isolation to induce schizophrenia on rodents. We will exclude studies that only used NMDA receptor antagonist to induce schizophrenia on rodents.

### 2.2. Outcomes

The primary outcome of this review will be cognitive impairments caused by schizophrenia, down- or upregulation of different genes involved in schizophrenia disorder, and neurochemical and structural changes related to schizophrenia disorder. The severity of positive, negative and cognitive symptoms will be evaluated. The gender effect will also be investigated by comparing the response of both male and female rodents in the “dual hit” model.

### 2.3. Inclusion Criteria

Studies will be included in this review based on the following criteria:

All studies that used the “dual hit” rodent model (post-weaning social isolation and NMDA receptor antagonist) to induce schizophrenia will be included. All published studies will be included regardless of sample size, gender, or year of publication; studies in other languages will only be included if they can be translated into English using Google translator.

### 2.4. Exclusion Criteria

We will exclude all non-rodent studies, editorials, letters, and case studies. All unpublished animal studies will also be excluded.

### 2.5. Information Sources

Literature search strategies will be developed using medical search headings (MeSH). The MEDLINE (PubMed), EMBASE, and Google Scholar databases will be used to search for published studies electronically. All databases will be searched from inception until 28 February 2021, with the literature search limited to the English language and rodent subjects. To confirm literature saturation, we will scan the reference list of the included studies. We will search the author’s individual files to ensure that all the relevant materials have been captured. Lastly, we will circulate a bibliography of the included articles to the systematic review team, as well as to the schizophrenia experts identified by the team.

### 2.6. Search Strategy

The review will be based on quantitative studies; no study design, language, or date limits will be applied to the search. Studies in languages other than English that can be translated sufficiently using Google translate will be included. The literature search will be conducted by two independent authors (Khanyiso Bright Shangase and Thabo Magwai), and the third author (Fredrick Otieno Oginga) will be consulted for arbitration. The search will be based on the following keywords and rodent subject headings: “schizophrenia”, “social isolation”, “NMDA receptor antagonists”, and “animal models”.

### 2.7. Interventions

Of interest are interventions that involve inducing schizophrenic symptoms using a “dual hit” rodent model (post-weaning social isolation and NMDA receptor antagonist). Most laboratory studies socially isolate rat and mouse pups by housing them individually from the first day of weaning from their dams, usually from PND21 to PND28. Depending on the individual researcher or study, the period of isolation can vary from 3 to 12 weeks, and the effects of social isolation depend on the period that the animals were isolated. There are many available NMDA receptor antagonists that researchers use to induce schizophrenia on rodents, and some antagonists are stronger than others. Different researchers use different NMDA receptor antagonists and different dosages to induce schizophrenia, depending on what they want to achieve in their individual studies.

### 2.8. Study Records

Results from the literature search will be uploaded to an internet-based software programme that eases collaboration among reviews during the study selection process, termed distiller systematic review (DSR). All references will be imported into the one single endnotes library version X7. Our team will develop and test screening questions and forms for level 1 and level 2 assessments based on the inclusion and exclusion criteria. Citation abstracts and full-text articles will be uploaded along with screening questions to the DSR. Before the formal screening process, the calibration screening exercise will be conducted to pilot and refine the screening questions. Before the start of the review, we will provide training to new team members not familiar with the DSR software and the content area.

### 2.9. Selection Process

The screening studies process will be performed by two independent authors (Khanyiso Bright Shangase and Thabo Magwai) to avoid any inconsistencies in terms of the eligibility of studies. First, studies will be screened by title, abstract, keywords and synonyms followed by identifying the full-text articles. Should any disagreements arise between two authors, a third author (Fredrick Otieno Oginga) will screen such studies, and an agreement will be reached through discussion. We will seek additional information from study authors where necessary to resolve questions about eligibility. All excluded studies will be listed in a table, and the reason for exclusion will be recorded. The PRISMA-flowchart will be prepared to document the final selection process. All selected or included studies will be subjected to data collection, critical appraisal, risk, and quality assessment.

### 2.10. Data Extraction

Standardised forms and a thorough instruction guidebook will be used to advise specific adaptations to an online data abstraction program DSR. Khanyiso Bright Shangase and Thabo Magwai will extract data, independently and in duplicate, from each eligible study to ensure that there is consistency between reviews. A calibration exercise will be run before the start of the review. The third author (Fredrick Otieno Oginga) will intervene for arbitration should any disagreements arise. The author and year of publication, country, sample size, study design, rodent characteristics (strain, age and gender), period of isolation, NMDA receptor antagonist used and dosage, types of control used, symptoms severity and treatment of symptoms, if included, will be recorded. The main authors of the studies will be contacted where inadequate data are provided to acquire enough information. Individual studies may consist of multiple treatment groups, such as different dosages of NMDA receptor antagonist or different social isolation periods. To prevent the possibility of introducing bias caused by multiple statistical comparisons with one control group, groups from various arms of study will be combined into a single group.

### 2.11. Risk of Bias and Quality Assessment

The Downs and Black checklist will be used to test the quality and biases of included studies [14]. The checklist is made up of 4 domains: reports of bias, external validity, internal validity, and selection bias. The checklist comprises 27 questions, with a maximum score of 27. The Downs and Black score is divided into 4 categories: excellent (26–27), good (20–25), fair (15–19), and poor (0–14) [15]. For all included studies, two independent authors will evaluate the quality of each study (Khanyiso Bright Shangase and Thabo Magwai). In case of disagreements, authors will discuss such studies, and the third author (Fredrick Otieno Oginga) will then adjudicate.

### 2.12. Publication Bias and Quality of Cumulative Evidence

We will test if the selective reporting of outcomes is present (outcome reporting bias), and the presence of small sample bias in the published literature will be evaluated by comparing the fixed effect estimate with the random effect model. The random effect estimate of interventions is mostly favoured versus the fixed effect estimate in the presence of small sample bias. In the case of 20 studies or more, risk rational or mean difference will be used as effective measures for a meta-analysis; furthermore, funnel plots will be produced, and Egger’s regression test will be used to measure publication bias. Studies will be sub-grouped according to geographic location and methodological quality. The quality of evidence for the primary outcomes will be evaluated using the grading of recommendation assessments, development, and evaluation (GRADE) tool [16].

### 2.13. Data Synthesis

If the design and comparator are sufficiently homogenous for all studies, a meta-analysis will be conducted using a random-effect model. Dichotomous outcomes will be measured by using a risk ratio (RR) with a 95% confidence interval (CI). Previously, it has been shown that RR is more intuitive than odds ratios (OR). Sometimes, clinicians tend to interpret OR as RR, which results in an overestimation of the effect. Weighted means differences (with 95% CI) will be used to analyse continuous data; in the event of different measurements, standardised mean differences (95% CI) will be used. A descriptive presentation will be used for skewed and non-quantitative data. All included data will be tested in order to determine the unit of randomisation and to determine if this unit of randomisation is dependable with the unit of analysis. Issues of interest in the analysis of studies with a non-standard design, such as studies with multiple treatment groups, cluster randomised trials, and cross-over trials, will be addressed. The interclass correlation co-efficient will be adopted to change the results according to the methods explained in the Cochrane Handbook for systematic reviews of intervention for cluster randomised trials. For a cross-over trial, only data from the first phase will be used and guided by the Cochrane Heart Group. For studies with more than two treatments, the additional treatment arm will be presented. A sensitive analysis will be conducted, and the heterogeneity in the randomisation unit will also be acknowledged.

If there are missing data, we will contact the study’s original authors to obtain and retrieve the missing data. Important numerical data will be thoroughly appraised. If the missing data are not retrieved, a linear mixed-effect model will be applied. We will also test for study heterogeneity by specifically looking at animal factors among trials (strain, age, randomisation concealment, blinding of outcome assessment, losses to follow-up, treatment type, and co-interventions). Statistical heterogeneity will be calculated using the *Chi2* test (significant level: 0.1) and *I2* statistic (0% to 40% might not be important; 30% to 60% might represent moderate heterogeneity; 50% to 90% might represent substantial heterogeneity; and 75% to 100% considerable heterogeneity) [17]. If a high level of heterogeneity is observed among the trials (*I2* > 50% or *p* < 0.1), the study design and the characteristics in the included studies will be analysed. The sensitivity analysis and subgroup analysis will be used to try to define and pinpoint the exact source of heterogeneity. If observed heterogeneity is *I2* > 50% or *p* < 0.1, the random effect model will be used; if heterogeneity is substantial, a meta-analysis will not be performed; instead, a qualitative narrative summary will be performed.

### 2.14. Confidence in Cumulative Estimates

Grading of recommendations assessment, development, and evaluation working group methodologies will be used to evaluate the quality of evidence for all outcomes. There are four domains of risk of bias: publication bias, consistency, directness, and precision. The quality of evidence will be tested across all these domains. The quality will be arbitrated at a high level: more research is improbable to change our confidence in the estimate of effect. This will be followed by a moderate level: more research is likely to have an important influence on our confidence in the estimate of effect and may change the estimate. Next, is low level: more research is likely to have an essential influence on our confidence in the estimate of effect and may change the estimate. Finally, a level of very low: very uncertain about the estimate of effect. The quality of evidence for the primary outcomes will be evaluated using the grading of recommendation assessments, development, and evaluation (GRADE) tool.

## 3. Discussion

Schizophrenia is a severe neuropsychiatric disorder affecting approximately 1% of the population globally [4,18]. The symptoms are divided into three groups: positive (conceptual disorganization, delusions, hallucinations and thought disorder), negative (anhedonia, motivation, avolition, poverty of thought, social withdrawal and poverty of speech) and cognitive symptoms (attention deficits, cognitive flexibility and working memory) [19,20]. Negative symptoms and cognitive impairments are resistant against current antipsychotic treatment. Most animal models of schizophrenia tend to replicate characteristics of positive symptoms of schizophrenia, thus neglecting the important aspect of negative symptoms [4]. Drug-induced models (ketamine, MK-801, and PCP) have been commonly used to replicate negative symptoms. In contrast, the developmental model (post-weaning social isolation) has been shown to reproduce positive symptoms [5,7,21]. To better understand and manage schizophrenia, it is imperative to develop a model that can reproduce the pathological state and most of the symptoms (both negative and positive), if not all. This systematic review will be the first to critically analyse and investigate the already available data on a schizophrenia rodent “dual hit” model. In order to improve the model, one needs to critically review the already available studies on a “dual hit” model of schizophrenia and identify gaps that need to be filled such that the specificity of the model can be improved. We propose that the results of this systematic review will contribute to the development of a more efficient model of schizophrenia that will be able to better characterize all its symptoms and that will accelerate the discovery of a new active treatment.

## Data Availability

Not applicable.
